# Supplementary material for: Contact-number-driven virus evolution: A multi-level modeling framework for the evolution of acute or persistent RNA virus infection
Source: PLoS Comput Biol. 2023 May 30;19(5):e1011173. doi: 10.1371/journal.pcbi.1011173 (PMC10256155; doi:10.1371/journal.pcbi.1011173)
Supplement: S1 Text — Table A. Parameters used for virus infection dynamics in the main text. Table B. Parameters used in GA Algorithm. Table C. Parameters used for the network models. Fig D. Relation between duration of infectious period and peak viral load. Fig E. Evolution of virus on a contact history generated from the Barabási-Albert scale free network model. Fig F. Evolution of virus on a contact history generated from the Watts-Strogatz small world network model. Fig G. Virus evolution in various scenarios of contact history. Fig H. Different function types in the duration of infectious period assumed to depend on the cumulative viral load. Fig I. Virus evolution in various scenarios of contact history where a monotonically decreasing linear relationship is assumed between the duration of the infectious period and the cumulative viral load. Fig J. Virus evolution in various scenarios of contact history where a monotonically decreasing exponential relationship is assumed between the duration of the infectious period and the cumulative viral load (DOCX) [file pcbi.1011173.s001.docx]

**Supplementary Information (S1 text)**

**Contact-number-driven virus evolution: a multi-level modeling framework for the evolution of acute or persistent RNA virus infection**

Junya Sunagawa^1^, Ryo Komorizono^2^, Hyeongki Park^3^, William S Hart^4^, Robin N. Thompson^5,6^, Akiko Makino^2,7^, Keizo Tomonaga^2,7,8,†^, Shingo Iwami^3,9,10,11,12,13†,*^ and Ryo Yamaguchi^1,14*^

^1^Department of Advanced Transdisciplinary Science, Hokkaido University, Sapporo, Hokkaido, Japan. ^2^Laboratory of RNA Viruses, Department of Virus Research, Institute for Life and Medical Sciences (LiME), Kyoto University, Kyoto, Japan. ^3^interdisciplinary Biology Laboratory (iBLab), Division of Natural Science, Graduate School of Science, Nagoya University, Nagoya, Japan. ^4^Mathematical Institute, University of Oxford, Oxford, United Kingdom. ^5^Mathematics Institute, University of Warwick, Coventry, United Kingdom. ^6^Zeeman Institute for Systems Biology and Infectious Disease Epidemiology Research, University of Warwick, Coventry, United Kingdom. ^7^Laboratory of RNA Viruses, Graduate School of Biostudies, Kyoto University, Kyoto, Japan. ^8^Department of Molecular Virology, Graduate School of Medicine, Kyoto University, Kyoto, Japan. ^9^Institute of Mathematics for Industry, Kyushu University, Fukuoka, Japan. ^10^Institute for the Advanced Study of Human Biology (ASHBi), Kyoto University, Kyoto, Japan. ^11^Interdisciplinary Theoretical and Mathematical Sciences Program (iTHEMS), RIKEN, Saitama, Japan. ^12^NEXT-Ganken Program, Japanese Foundation for Cancer Research (JFCR), Tokyo, Japan. ^13^Science Groove Inc., Fukuoka, Japan 8100041. ^14^Department of Zoology & Biodiversity Research Centre, University of British Columbia, Vancouver, British Columbia, Canada.

^1^To whom correspondence may be addressed.

Email: [iwami.iblab@bio.nagoya-u.ac.jp](mailto:iwami.iblab@bio.nagoya-u.ac.jp) (S.I.) and [ryamaguchi@sci.hokudai.ac.jp](mailto:ryamaguchi@sci.hokudai.ac.jp) (R.Y.).

Address: Furo-cho, Chikusa Nagoya 464-8602, Japan (S.I.), Phone: +81-52-789-2992 (S.I.) and Sapporo, Hokkaido 060-0810, Japan (R.Y.), Phone: +81-11-706-2659 (R.Y.)

**Table A**

**Parameters used for virus infection dynamics in the main text**

| parameter | value |
| --- | --- |
| Number of uninfected target cells at initial time, $\boldsymbol{T}\left( \boldsymbol{t=0} \right)$ | $1000$ |
| Number of infected target cells at initial time, $\boldsymbol{I}\left( \boldsymbol{t=0} \right)$ | $0$ |
| Amount of infectious virus at initial time, $\boldsymbol{V}_{\boldsymbol{i}}\left( \boldsymbol{t=0} \right)$ | $1$ |
| Amount of noninfectious virus at initial time, $\boldsymbol{V}_{\mathbf{non}}\left( \boldsymbol{t=0} \right)$ | $0$ |
| Initial number of uninfected cells, $\boldsymbol{\lambda}$ | $10$ |
| Rate of virus infection, $\boldsymbol{\beta}$ | $0.0001$ |
| Death rate of noninfectious cells, $\boldsymbol{\mu}$ | $0.01$ |
| Death rate of infectious cells, $\boldsymbol{\delta}$ | $\delta_{\max}p/\left( p+p_{50} \right)$ |
| Maximum value of $\boldsymbol{\delta}$, $\boldsymbol{\delta}_{\mathbf{max}}$ | $1$ |
| Viral production rate satisfying $\boldsymbol{\delta}_{\mathbf{max}}\boldsymbol{/2}$, $\boldsymbol{p}_{\boldsymbol{50}}$ | $1000$ |
| Clearance rate of the virus, $\boldsymbol{c}$ | $10$ |
| Proliferative ability of virus, $\boldsymbol{p}$ | [0, 100, 200, …, 9900] (100 points in total) |
| Accuracy of virus replication, $\boldsymbol{\varepsilon}$ | [0.0, 0.01, 0.02, …, 0.99] (100 points in total) |
| Maximum duration of the infectious period, $\boldsymbol{D}_{\mathbf{max}}$ | $1000$ |
| Viral load at which the duration is half of its maximum, $\boldsymbol{V}_{\boldsymbol{50}}$ | $1.0\times{10}^{6}$ |
| Steepness at which duration decreases with increasing viral load, $\boldsymbol{D}_{\boldsymbol{k}}$ | $7.5$ |
| Basilar probability of infection immediately, $\boldsymbol{b}$ | $9.8\times{10}^{-8}$ |
| r-fold increase in infectiousness for each 10-fold increase in infectious viral load, $\boldsymbol{r}$ | $25$ |

**Table B**

**Parameters used in GA Algorithm**

| parameter | value |
| --- | --- |
| The size of the population | $100$ |
| The number of iterations | $100$ |
| The number of elitism | $2$ |
| The probability of selection | $1.0$ |
| The probability of crossover | $0.7$ |
| The probability of mutation | $0.1$ |
| The pre-defined range of $\boldsymbol{p}$ in mutation | $200$ |
| The pre-defined range of $\boldsymbol{\varepsilon}$ in mutation | $0.2$ |

**Table C**

**Parameters used for the network models**

| model | parameter | value |
| --- | --- | --- |
| BA | number of nodes, $n$ | $100$ |
|  | number of edges to attach from a new node to existing nodes, $m$ | $1$ |
| WS | number of nodes, $n$ | $100$ |
|  | nearest nodes which are connected, $k$ | $2$ |
|  | the probability of rewiring each edge, $p$ | $0.5$ |

BA: Barabási-Albert scale-free network model

WS: Watts-Strogatz small world network model


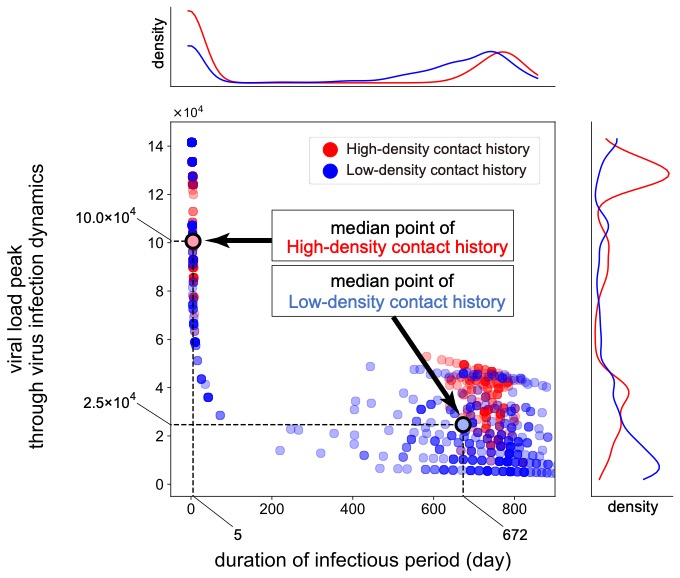


**Fig D. Relation between duration of infectious period and peak viral load.** In each independent iteration, viruses evolve for the duration of the infectious period and the peak viral load depending on the contact history: high-density contact history (red) and low-density contact history (blue), respectively. The black arrows correspond to the median values representing the evolutionary endpoints. The parameter sets of shape and scale used are as follows: $\left( k, \theta\right)=\left( 1000, 0.002 \right)$ for high-density contact history, and $\left( k, \theta\right)=\left( 0.12, 10 \right)$ for low-density contact history, respectively. For both contact histories, the distributions of duration of infectious period are illustrated as kernel plots above the main figure. Also shown to the right of the main figure are the distributions of viral load peak through virus infection dynamics. For the high-density contact history, virus evolution results in a short duration of infectious period (5 days) with a high viral load ($1.0\times{10}^{5}$). In contrast, for low-density contact history, virus evolution results in a long duration of infectious period (672 days) with a low viral load ($2.5\times{10}^{4}$). 500 simulations were run for each contact history parameter set.


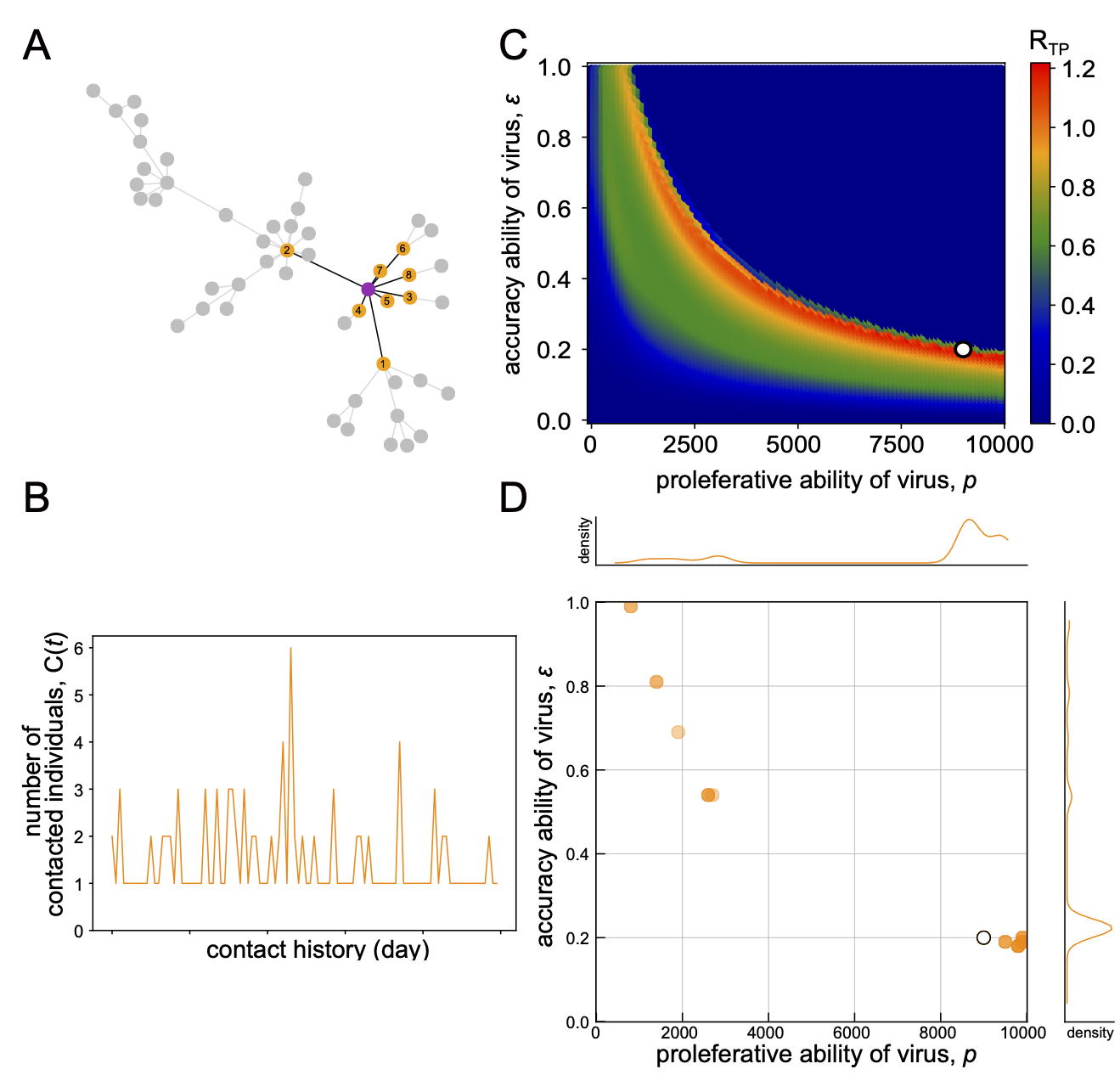


**Fig E. Evolution of virus on a contact history generated from the** **Barabási-Albert scale-free network model.** An example graph of the BA model is illustrated in **(A)**. Contact numbers are calculated by counting the number of nodes that are connected to a single node chosen at random (purple). In this example, the contact number is 8 (orange). Iterating this process generates a contact history. An example of a given contact history is plotted in **(B)** (only 100 days is shown). Optimal parameter combinations ($p$ and $\varepsilon$) to increase $R_{TP}$are calculated for a given contact history generated from the BA model in **(C)**. Color represents the degree of $R_{TP}$: blue corresponds to lower values of $R_{TP}$, and red corresponds to higher values of $R_{TP}$. The white dot represents the optimal point that increases $R_{TP}$ in a single simulation (*see* the corresponding white dot in **(D)**). Using the same parameter set, 50 optima based on independent contact histories are calculated in **(D)**. The kernel plots illustrated at the top and right of the figure are the distribution of $p$ and $\varepsilon$, respectively.


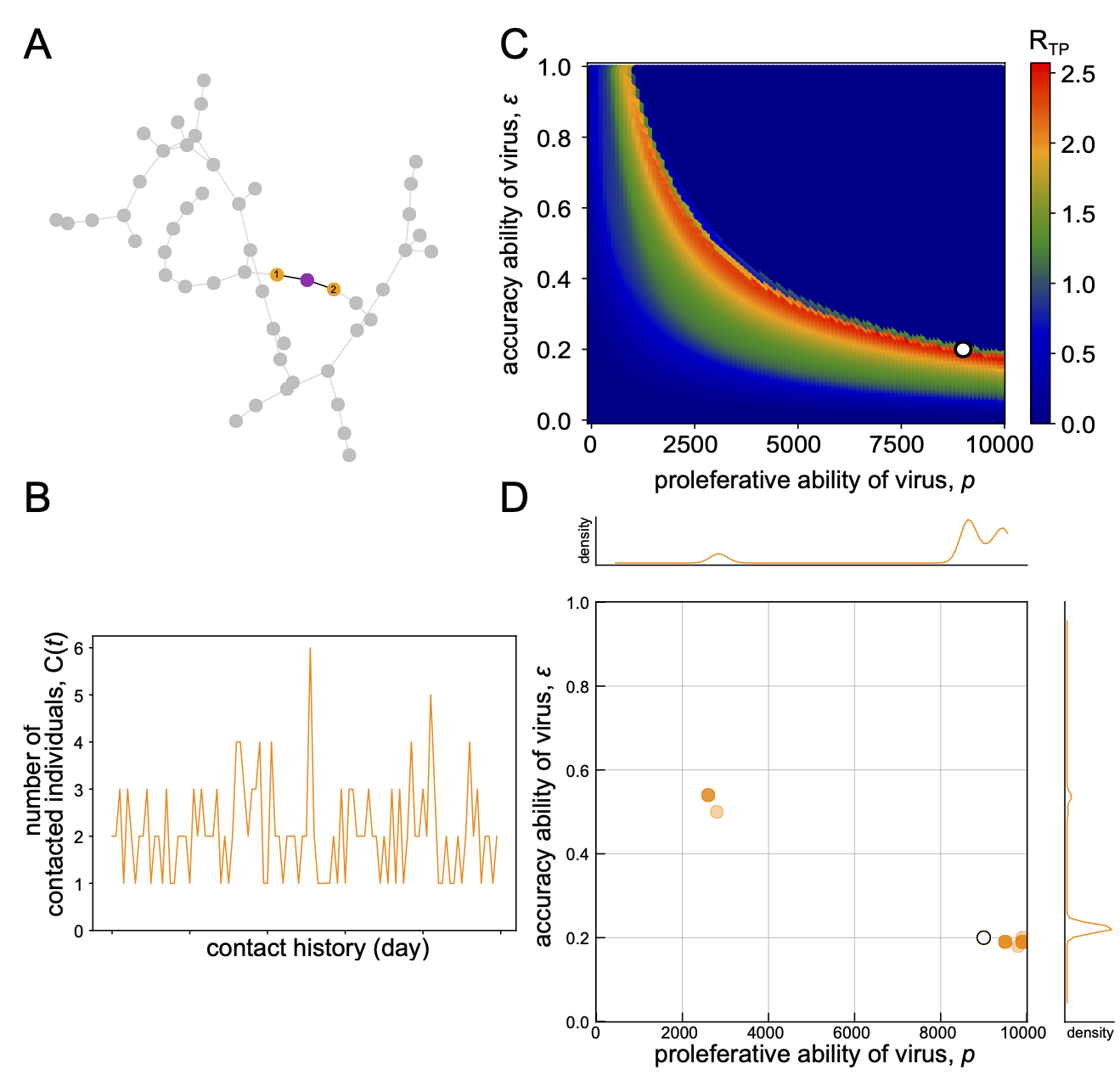


**Fig F. Evolution of virus on a contact history generated from the Watts-Strogatz small world network model.** An example graph of the WS model is illustrated in **(A)**. Contact numbers are calculated by counting the number of nodes that are connected to a single node chosen at random (purple). In this example, the contact number is 2 (orange). Iterating this process generates a contact history. An example of a given contact history is plotted in **(B)** (only 100 days is shown). Optimal parameter combinations ($p$ and $\varepsilon$) to increase $R_{TP}$ are calculated for a given contact history generated from the WS model in **(C)**. Color represents the degree of $R_{TP}$: blue corresponds to lower values of $R_{TP}$, and red corresponds to higher values of $R_{TP}$. The white dot represents the optimal point that increases $R_{TP}$ in a single simulation (*see* the corresponding white dot in **(D)**). Using the same parameter set, 50 optima based on independent contact histories are calculated in **(D)**. The kernel plot illustrated at the top and right of the figure are the distribution of $p$ and $\varepsilon$, respectively.

**

**

**Fig G. Virus evolution in various scenarios of contact history.** Each row represents a single parameter set of ($\boldsymbol{\delta}_{\boldsymbol{max}}$ and $\boldsymbol{p}_{\boldsymbol{50}}$), that is **(A)**, **(B)**, **(C)** for (1.0, 500), (1.0, 2000), and (2.0, 2000), respectively. A total of 441 contact history patterns are explored for each parameter set of ($\boldsymbol{\delta}_{\boldsymbol{max}}$ and $\boldsymbol{p}_{\boldsymbol{50}}$) to determine the vial evolution to optimize $R_{TP}$ in a given environmental scenario. We conducted a total of 500 independent iterations for each parameter combination of $k$ and $\theta$ as well as in **Fig.3** in the main text. Numbers 1, 2, 3, and 4 in the panels of viral load are the corresponding dynamics illustrated as white dots in the columns of $p$, $\varepsilon$, the duration of infectious period, and $R_{TP}$, respectively. In the panels of $R_{TP}$, the vertical dashed line separates the area of mean contact number by an index, $R_{TP}={10}^{0} \left( =1 \right)$ described by the horizontal dashed lines. The corresponding dashed line is also illustrated in the panels of $p$, $\varepsilon$, and the duration of infectious period.

**
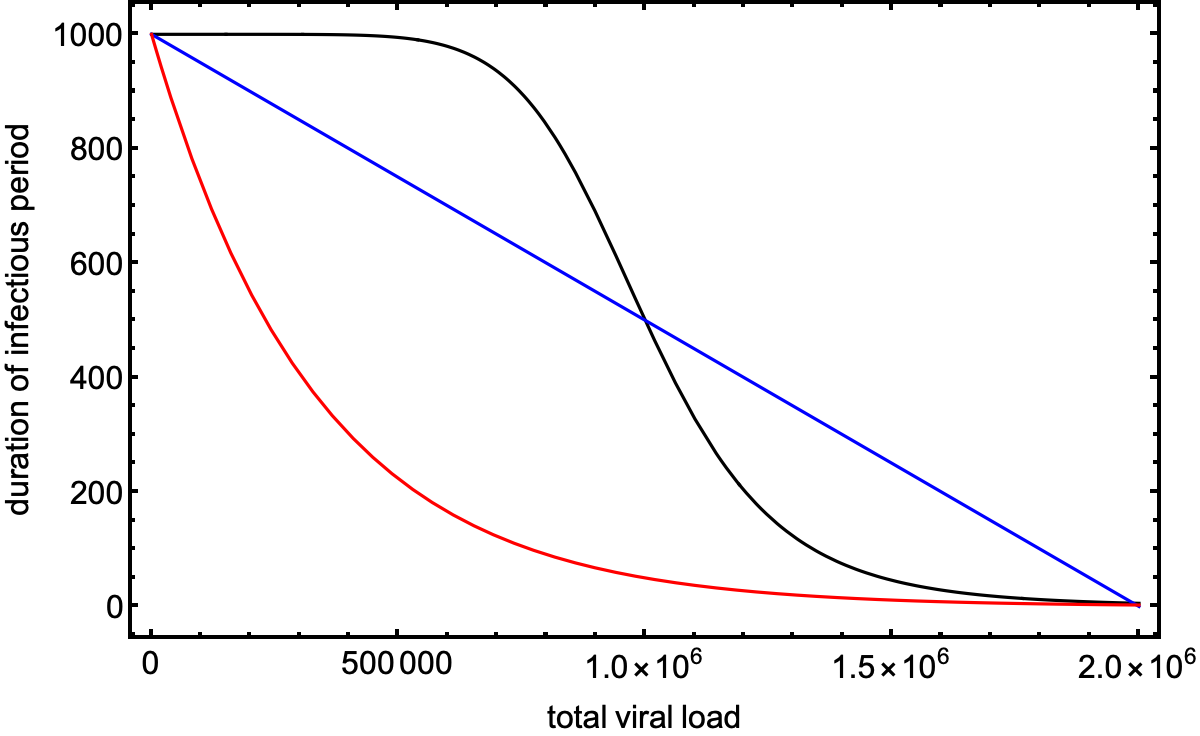
**

**Fig H. Different function types in the duration of infectious period assumed to depend on the cumulative viral load.** The black line represents the sigmoidal relationship calculated by Eq.(5) shown in the main text, and the function shape is exactly the same as that used throughout the paper (i.e., using the parameters provided in **Table A in S1 text**). For elucidating the monotonically decreasing relationship, we define the linear (blue line) and the exponential (red curve) relationship as follows: $D_{\max}-mV_{\mathrm{total}}$ and $D_{\max}\exp(-nV_{\mathrm{total}})$, respectively. We have assumed that the maximum duration of the infectious period is the same (1000) among all the functions. For testing robustness in the results of the current model using the linear and exponential relationship, see the following **Fig I** and **J in S1 text**. Parameters include $m=5.0\times{10}^{-4}$ and $n=3.0\times{10}^{-6}$.





**Fig I. Virus evolution in various scenarios of contact history where a monotonically decreasing linear relationship is assumed between the duration of the infectious period and the cumulative viral load.** A total of 441 contact history patterns are explored to determine the viral evolution to an optimal $R_{TP}$ in a given environmental scenario. 500 iterations of each parameter combination of $k$ and $\theta$ are conducted to determine the optimized parameter set of $p$ and $\varepsilon$ characterizing final virus infection dynamics (i.e., different viral infection phenotypes). The median of the optimized parameter set of $p$ and $\varepsilon$ based on the 500 simulation runs is shown against the mean contact number of histories (with logarithmic increase) determined by each parameter set of $k$ and $\theta.$ The combination of $p$ and $\varepsilon$ is plotted in **(A)** and **(B)**, separately. Colors represent the degree of the mean contact number: blue corresponds to a low mean contact number, whereas red corresponds to a high mean contact number. How virus infection dynamics vary with the mean contact number is shown in (**C**). Several examples of the virus infection dynamics are shown to represent the difference in the infectious period and the peak viral load. Numbers 1, 2, 3, and 4 in **(C)** are the corresponding dynamics illustrated as white dots in **(A)**, **(B)**, **(D)**, and **(E)**, respectively. The duration of the infectious period and $R_{TP}$ by the mean contact number are plotted in (**D**) and (**E**), respectively. In **(E)**, the vertical dashed line separates the area of mean contact number by an index, $R_{TP}={10}^{0}\left( =1 \right)$. The corresponding dashed line is also illustrated in **(A)**, **(B)**, and **(D)**. Concerning the actual function shape for the duration of infectious period, see **Fig H in S1 text**.





**Fig J. Virus evolution in various scenarios of contact history where a monotonically decreasing exponential relationship is assumed between the duration of the infectious period and the cumulative viral load.** A total of 441 contact history patterns are explored to determine the viral evolution to an optimal $R_{TP}$ in a given environmental scenario. 500 iterations of each parameter combination of $k$ and $\theta$ are conducted to determine the optimized parameter set of $p$ and $\varepsilon$ characterizing final virus infection dynamics (i.e., different viral infection phenotypes). The median of the optimized parameter set of $p$ and $\varepsilon$ based on the 500 simulation runs is shown against the mean contact number of histories (with logarithmic increase) determined by each parameter set of $k$ and $\theta.$ The combination of $p$ and $\varepsilon$ is plotted in **(A)** and **(B)**, separately. Colors represent the degree of the mean contact number: blue corresponds to a low mean contact number, whereas red corresponds to a high mean contact number. How virus infection dynamics vary with the mean contact number is shown in (**C**). Several examples of the virus infection dynamics are shown to represent the difference in the infectious period and the peak viral load. Numbers 1, 2, 3, and 4 in **(C)** are the corresponding dynamics illustrated as white dots in **(A)**, **(B)**, **(D)**, and **(E)**, respectively. The duration of the infectious period and $R_{TP}$ by mean contact number are plotted in (**D**) and (**E**), respectively. In **(E)**, the vertical dashed line separates the area of mean contact number by an index, $R_{TP}={10}^{0}\left( =1 \right)$. The corresponding dashed line is also illustrated in **(A)**, **(B)**, **(D)**. Concerning the actual function shape for the duration of infectious period, see **Fig H in S1 text**.
