# Supplementary material for: Contact-number-driven virus evolution: A multi-level modeling framework for the evolution of acute or persistent RNA virus infection
Source: PLoS Comput Biol. 2023 May 30;19(5):e1011173. doi: 10.1371/journal.pcbi.1011173 (PMC10256155; doi:10.1371/journal.pcbi.1011173)
Supplement: Attachment — Submitted filename: PBP_revision_R.pdf [file pcbi.1011173.s002.pdf]

<u>Response to reviewer comments</u>

Junya Sunagawa, Ryo Komorizono, Hyeongki Park, William S. Hart, Robin N. Thompson, Akiko Makino, Keizo Tomonaga, Shingo Iwami and Ryo Yamaguchi

February 18, 2023

We would like to thank the reviewers for their thorough reading of the manuscript and their excellent comments and suggestions. In this letter, we present the reviewers' comments in black and provide our responses in red. All the changes made in response to the reviewers' comments in PCOMPBIOL-D-22-01431 have been incorporated in the revised version of the manuscript. We believe that the manuscript has been considerably improved by these changes. All coauthors agreed with these changes.

Reviewer #1:

The authors present a study examining the evolution of chronicity (the ability to cause chronic versus acute infections) in response to varying host contact patterns. Focusing on the case study of viruses, the authors construct a model linking within-host dynamics to variable contact rate patterns, where new hosts are contacted at a rate that either has a high mean and low variance or a low mean and large variance (subsequently extended to a range of means and variances). They then use simulations to determine the viral proliferation rate and accuracy that maximize R0, the number of hosts infected over the lifespan of the primary infection. The results suggest a tradeoff between proliferation rates and replication accuracy, where the optimal combination depends on contact rate patterns. When hosts contact each other at a rate with a high mean, that selects for fast proliferating viruses with low accuracy, while low rates of contact select for slow proliferating viruses with high accuracy. These results suggest that host contact patterns can exert a strong influence over traits expressed within the host, including proliferation rates.

The question of what drives the evolution of chronicity is a very important one, and the model used by the authors is simple in the best way. There are no extraneous details in the model structure (like the inclusion of immunity) that would make it difficult to understand the results. The figures are visually appealing and easy to understand, and I appreciate that the authors repeated their analysis for a large range of mean/variance relationships. While I admire the clarity of the approach, there are a few areas where the MS would benefit from revision:

Reply to Comments:

Thank you for encouraging us through your positive evaluation and for your constructive comments to improve the quality of our manuscript. In this version, we reply to all the major and minor comments as follows.

【Major comments】

1. The authors could do much more to place this study in the context of previous work in this area. Past work has examined the evolution of replication rates while allowing infection duration to depend on viral load (King et al. 2009 Am Nat), which may necessitate rewording lines 200-202. That study and subsequent ones (Shrestha et al. 2013 Theoret Ecol) suggest important limitations to the idea that natural selection will always maximize R0, and

those limitations would be worth discussing further in this MS (such as lines 104-106). There's also a wealth of literature on the evolution of acuteness especially in the context of measles that would be good to discuss here (e.g., Gunning & Wearing 2013 Ecol Lett). Referencing this body of work—and making the unique contributions of this study very clear—would greatly enhance its impact.

Reply to comment 1:

Thank you for suggesting the relevant literature, which strongly guided our revisions. We have substantially revised our introduction (page 5, lines 109-110) and discussion (page 11, lines 223-237) by adding the suggested papers.

2. I realize that putting the methods after the results is part of this journal's format, but more methods need to be outlined at the beginning of the results to make sense of what follows. I'd suggest adding a panel to Fig. 1 to illustrate the within-host model structure and make it obvious visually that the rate of transmission varies with viral load. One idea would be to arrow size constant within ta (and within tb and tc) but varying arrow size with viral load to emphasize that both transmission rates per contact and contact rates are changing over the course of infection, but only transmission rates per contact depend on viral load. Some might misinterpret Fig. 1A as showing that host contact rates vary with viral load (not an unreasonable assumption, but not what this model is doing). It would also be helpful to give R0 for this example (R0=4, if I'm reading the figure example correctly) so that readers can be sure they are following along.

Reply to comment 2:

Thank you very much for pointing this out. We have revised the corresponding figure (see our new **Fig.1**) and have revised some explanations to clarify our modelling assumptions before presenting the main results.

3. It is not clear to me why proliferation rates tradeoff with accuracy. The death rate of infected cells increases (but saturates) with increasing p, a cost of replicating faster. But why don't viruses always evolve to maximize accuracy? My best guess is that fast proliferation, when combined with high accuracy, depletes target cell abundance and causes disfavorable fluctuations in viral

load (i.e., causing low transmission rates that reduce the number of new hosts infected). If that guess is correct, it would be good to make that clearer, since an implicit tradeoff that arises from the biologically reasonable assumptions is (in my opinion) much more interesting than a tradeoff that was explicitly assumed at the outset.

Reply to comment 3:

We have added a new paragraph to clarify this issue in the results (pages 9-10, lines 194-213). Many RNA viruses have high mutation rates. This is due to the lack of proofreading ability of the viral RdRp. Referring to previously published virological experiments, recombinant viruses with higher fidelity were reported to be less virulent in vivo and to have a reduced growth rate. These high-fidelity viruses also differed significantly in the viral distribution in vivo [1, 2]. Genetic diversity through mutation is the driving force to adapt to the intra-host environment. Furthermore, in vivo and in vitro experiments have shown that there is a trade-off between the replication speed and fidelity of RdRp [3]. WJ Fitzsimmons et al. would be a suitable candidate for an answer to your question. Isolation of mutant viruses with higher mutation rates was observed when viruses with faster replication speed were systematically selected for. From the above reports, it is difficult to achieve both high fidelity maximization and high proliferation in RNA viruses, and proliferation is compromised by reduced fitness due to reduced genetic diversity.

4. It would also be good to discuss a key counterexample, why HIV has been able to persist as a chronic infection despite the results in this MS suggesting that it should have evolved to be a faster-proliferating virus. My sense is that HIV violates a key assumption in the present model, that inaccurate replication does not impact infection duration. Mutations within the host could instead lead to variants that are better able to evade the immune system, despite their reduced ability to transmit (Lythgoe et al. 2017 Trends Microbiol). Omitting mention of this prominent counterexample might come across to readers as cherry-picking only case studies that agree with the model results.

Reply to comment 4:

We have added a paragraph to clarify this issue (pages 9-10, lines 194-213). This study only refers to RNA viruses, not retroviruses. HIV-1 is a retrovirus

with a life cycle that differs significantly from that of common RNA viruses [4]. HIV-1 produces viral “DNA” by its own reverse transcriptase after entering CD4-positive cells. This viral DNA is then inserted into the host chromosome by viral integrase and merges with the host genome as a provirus. Viral RNA transcribed from the provirus is synthesized by RNA polymerase II (Pol II) in exactly the same way as mRNA encoded in host chromosomal DNA [5]. Thus, the evolution of HIV-1 has been influenced by this different life cycle from RNA viruses, in particular the replication of the viral genome by higher fidelity host polymerase, and it is inferred that it persists as a chronic disease.

【Minor comments】

5. Lines 255-256: Seasonal forcing in contact rates aren’t explicitly examined in this model, and it’s not clear how the results could be extrapolated to this case. Clarify or omit?

Reply to comment 5:

Thank you very much for this thought-provoking comment. Our model can be extended without loss of generality by assuming that the average value in the contact history is a periodic function. However, it is an interesting future question whether the virus will likely adapt to the importance of seasons with less contact or adapt to take advantage of seasons with more contact. We have added a brief explanation to clarify this point (page 13, lines 291-293).

6. Lines 303-308: This phrasing undercuts the value of this study without adding useful nuance for readers. I agree with the authors that including immunity wouldn’t change the conclusions, but leading the reader through that logic would serve the authors better here. Could the authors say more about their assumption that viral proliferation is limited by target cell availability (bottom-up control) rather than immunity (top-down control)? It strikes me that target cell availability will be a problem for viruses whether or not hosts mount a useful immune response. Likewise, if immunity reduces symptoms/negative health outcomes but does not strongly impede viral replication (as in COVID-19), then target cell limitation takes on even greater importance.

Reply to comment 6:

Thank you very much for this important point. As pointed out, our virus

dynamics model, Eqs.(1-4), is called a “target cell-limited model” and does not explicitly include “immune response”. Instead, the effect of the immune response is implicitly included in certain parameters, such as the death rate of infected cells and the virus infection rate. However, the virus's proliferation is still limited by target cell availability, regardless of whether the immune response is explicitly included or not. We have changed the paragraph to clarify this issue (page 15, lines 329-340).

7. Lines 323-326: It seems strange to discuss surveillance systems when the model has more relevance to contact rate interventions like social distancing.

Reply to comment 7:

We have changed this part to emphasize the importance of extracting the contact history of a host to predict/understand the evolution of future viruses (page 16, lines 357-358).

8. Supplemental text S1 and S2: I'm not sure why this text is relegated to the supplement. Might be worth including in the main text?

Reply to comment 8:

We have added these sections to the introduction (page 9, lines 217-236) and methods (page 21, lines 469-478), respectively.

Reviewer #2:

1. Throughout the paper, persistent viruses are characterized by low viral replication rate and decreasing evolutionary rate, which is not true in general. In particular in the introduction, they state "persistent infectious virus can coexist with the host by reducing the amount of progeny virus released from the infected cells, slowing down the replication rate, decreasing evolutionary rate, and evading the host's immune system". Viruses such as HCV, referred to as an example, and HIV actually have high viral production and evolutionary rate, using this to evade the immune response and be persistent virus, but also include latent reservoir which might be a very nuanced case to characterize as persistent low replication. Further discussion of this strategy for some persistent viruses and how it reconciles with their framework and results would be helpful. While the authors do acknowledge in the Discussion that antigenic escape, not considered in this study, may change the methods and results, it seems that they are discussing coevolution between hosts rather than viral evolution within host against immune response.

Reply to comment 1:

Thank you very much for your important comments. HCV and BDV, which have already been described in this study, are representative of persistent infectious RNA viruses [6]. Indeed, HCV has been reported to produce relatively large numbers of viral particles during in vivo infection. On the other hand, viral replication in infected cells is suppressed by microRNAs and host exonuclease, as shown in previous studies [7]. In addition, HCV, like other RNA viruses, accumulates mutations in the host and constitutes a quasispecies [8], but the evolutionary rate of HCV in vivo has been shown to be slower than expected, thus questioning the conclusion that HCV has a very high evolutionary rate [9-13].

Similarly, BDV, a persistent infectious RNA virus, has been reported to have a suppressed intracellular replication speed and a very slow evolutionary rate [14, 15]. The number of virus particles released from BDV-infected cells is also extremely low [16].

In addition to the above-mentioned typical persistent infectious RNA viruses, a similar phenomenon has been observed when acutely infectious viruses undergo a phenotypic shift to persistent infection. For example, when coronaviruses shift their phenotype to persistent infectivity through mutation

accumulation, translation efficiency and replication speed are markedly suppressed, and persistent infection is established [17, 18]. Coxsackieviruses also establish persistent infection through defective mutations at the terminus of the genome, which markedly suppresses replication speed and viral particle production [19]. Furthermore, it has been shown that persistently infected BVDV strains evolve at an extremely slower rate than acutely infected strains in vivo [20-23].

Therefore, it is difficult to generalize the properties of persistently infectious RNA viruses, but the characteristics defined in this study are indeed widely observed phenomena. However, a limitation of this study is that immune responses and antigenic escape within the host may alter the results.

2. There is a lack of clarity in how the parameter ¥epsilon is being utilized in the evolutionary modeling. First, the authors give nebulous definitions of ¥epsilon, saying one line 120, it "includes multiple-factors such as the accuracy of virion assembly, the rate of virus particle aggregation and others, in addition to the accuracy of replication which is the main factor. However the parameter is seemingly a simple probability of viral progeny being "infection capable" in Eq. 3-4. It also seems that ¥epsilon is a completely independent parameter, as there is no explicit mention of a tradeoff between ¥epsilon and viral production rate p. Nevertheless, the Results seem to suggest that the authors are assuming some kind of tradeoff between ¥epsilon and viral production rate p (the contour lines in p vs ¥epsilon fitness graphs shown in Fig. 1B, 2). They do have a tradeoff formula between parameters p and infected cell death rate ¥delta, but ¥epsilon is not in this formula. It would make sense to have all these tradeoffs, in particular without it, one would expect viruses to always optimize their fitness by maximizing accuracy at ¥epsilon=1. I suppose that the infection duration being a decreasing function of total viral load (Eq. 5) could be the tradeoff, but the authors need to clarify and discuss. Also, this assumption in Eq. 5 needs further discussion as it seems critical for the results.

Reply to comment 2:

We have added a clear explanation of the accuracy ability of virus (ε). To be clear, we did not make any assumptions about a trade-off between the accuracy ability of virus (ε) and the proliferative ability of virus (p), while it is

assumed that the death rate of virus-producing cells (δ) depends on the proliferative ability of virus (p) due to viral cytopathogenesis. In contrast, we assumed that the duration of the infectious period decreases with the cumulative viral load, which is a crucial assumption in our study. This is because epidemiological studies have shown that patients with severe disease and a short duration of infection tend to have a high viral load, while those with relatively mild disease tend to have a low viral load [24-26]. Under these biologically reasonable assumptions, the trade-off in the duration of infectious period, as shown in **Fig. 1B**, arises from our individual-level virus infection model, as described in Eqs.(1-4). To relax the assumption of the relationship by Eq.5, we have performed the calculations using other decreasing functions, such as linear and exponential functions, as our sensitivity analyses. A newly added **Fig.S6**, **S7**, and **S8** show that our current conclusion is quite robust regardless of the function shape.

3. The mutation rate or precise formulation used in genetic algorithm and evolutionary computations are not given. Also one might think of this being tied to accuracy of replication, which is one of the terms describing ¥epsilon above.

Reply to comment 3:

We have added an explanation to clarify this issue (page 13, lines 276-282). Please also see our new **Table S2**.

4. In Discussion, authors state "none of previous researches assumed the relationship between viral load and viral duration of infection". However, it should be noted that several works have considered immuno-epidemiological multi-scale models for evolution of pathogen with immune response in within-host model, which consider various forms of recovery rates that mechanisticly capture the relationship between viral load and duration of infection; e.g. "Gulbudak H, Cannataro VL, Tuncer N, Martcheva M (2017) Vector-borne pathogen and host evolution in a structured immuno-epidemiological system. Bull Math Biol 79(2):325–355".

Reply to comment 4:

Thank you for suggesting this relevant literature. In the revised version, we have tried to explain our appeal in a slightly toned-down way, citing this

literature. Please also see our answer to major comment #1 raised by reviewer #1.

References:

1. Pfeiffer JK, Kirkegaard K. Increased fidelity reduces poliovirus fitness and virulence under selective pressure in mice. PLoS pathogens. 2005;1(2):e11.
2. Vignuzzi M, Stone JK, Arnold JJ, Cameron CE, Andino R. Quasispecies diversity determines pathogenesis through cooperative interactions in a viral population. Nature. 2006;439(7074):344-8. doi: 10.1038/nature04388.
3. Fitzsimmons WJ, Woods RJ, McCrone JT, Woodman A, Arnold JJ, Yennawar M, et al. A speed–fidelity trade-off determines the mutation rate and virulence of an RNA virus. PLOS Biology. 2018;16(6):e2006459. doi: 10.1371/journal.pbio.2006459.
4. Soper A, Kimura I, Nagaoka S, Konno Y, Yamamoto K, Koyanagi Y, et al. Type I Interferon Responses by HIV-1 Infection: Association with Disease Progression and Control. Frontiers in Immunology. 2018;8.
5. Li R, Sklutuis R, Groebner JL, Romerio F. HIV-1 natural antisense transcription and its role in viral persistence. Viruses. 2021;13(5):795.
6. Randall RE, Griffin DE. Within host RNA virus persistence: mechanisms and consequences. Curr Opin Virol. 2017;23:35-42. Epub 20170317. doi: 10.1016/j.coviro.2017.03.001. PubMed PMID: 28319790; PubMed Central PMCID: PMCPMC5474179.
7. Li Y, Masaki T, Yamane D, McGivern DR, Lemon SM. Competing and noncompeting activities of miR-122 and the 5′ exonuclease Xrn1 in regulation of hepatitis C virus replication. Proceedings of the National Academy of Sciences. 2013;110(5):1881-6. doi: 10.1073/pnas.1213515110.
8. Geller R, Estada Ú, Peris JB, Andreu I, Bou J-V, Garijo R, et al. Highly heterogeneous mutation rates in the hepatitis C virus genome. Nature Microbiology. 2016;1(7):16045. doi: 10.1038/nmicrobiol.2016.45.
9. Raghwani J, Rose R, Sheridan I, Lemey P, Suchard MA, Santantonio T, et al. Exceptional Heterogeneity in Viral Evolutionary Dynamics Characterises Chronic Hepatitis C Virus Infection. PLOS Pathogens. 2016;12(9):e1005894. doi: 10.1371/journal.ppat.1005894.
10. Gray RR, Parker J, Lemey P, Salemi M, Katzourakis A, Pybus OG. The mode and tempo of hepatitis C virus evolution within and among hosts. BMC Evolutionary Biology. 2011;11(1):131. doi: 10.1186/1471-2148-11-131.
11. Ribeiro RM, Li H, Wang S, Stoddard MB, Learn GH, Korber BT, et al. Quantifying the diversification of hepatitis C virus (HCV) during primary infection: estimates of the in vivo mutation rate. 2012.

12. Nakamura F, Takeda H, Ueda Y, Takai A, Takahashi K, Eso Y, et al. Mutational spectrum of hepatitis C virus in patients with chronic hepatitis C determined by single molecule real-time sequencing. Scientific Reports. 2022;12(1):7083. doi: 10.1038/s41598-022-11151-6.
13. Tisthammer KH, Solis C, Orcales F, Nzerem M, Winstead R, Dong W, et al. Assessing in vivo mutation frequencies and creating a high-resolution genome-wide map of fitness costs of Hepatitis C virus. PLoS Genetics. 2022;18(5):e1010179.
14. Kinnunen PM, Palva A, Vaheri A, Vapalahti O. Epidemiology and host spectrum of Borna disease virus infections. J Gen Virol. 2013;94(Pt 2):247-62. Epub 20121205. doi: 10.1099/vir.0.046961-0. PubMed PMID: 23223618.
15. Staeheli P, Sauder C, Hausmann J, Ehrensperger F, Schwemmle M. Epidemiology of Borna disease virus. J Gen Virol. 2000;81(Pt 9):2123-35. doi: 10.1099/0022-1317-81-9-2123. PubMed PMID: 10950968.
16. Pauli G, Ludwig H. Increase of virus yields and releases of Borna disease virus from persistently infected cells. Virus Research. 1985;2(1):29-33. doi: https://doi.org/10.1016/0168-1702(85)90057-7.
17. Banerjee A, Subudhi S, Rapin N, Lew J, Jain R, Falzarano D, et al. Selection of viral variants during persistent infection of insectivorous bat cells with Middle East respiratory syndrome coronavirus. Scientific Reports. 2020;10(1):7257. doi: 10.1038/s41598-020-64264-1.
18. Ke T-Y, Liao W-Y, Wu H-Y. A leaderless genome identified during persistent bovine coronavirus infection is associated with attenuation of gene expression. PLoS One. 2013;8(12):e82176.
19. Chapman NM. Persistent enterovirus infection: little deletions, long infections. Vaccines. 2022;10(5):770.
20. Peterhans E, Bachofen C, Stalder H, Schweizer M. Cytopathic bovine viral diarrhea viruses (BVDV): emerging pestiviruses doomed to extinction. Vet Res. 2010;41(6):44. Epub 20100304. doi: 10.1051/vetres/2010016. PubMed PMID: 20197026; PubMed Central PMCID: PMCPMC2850149.
21. Hamers C, Lecomte C, Kulcsar G, Lambot M, Pastoret PP. Persistently infected cattle stabilise bovine viral diarrhea virus leading to herd specific strains. Vet Microbiol. 1998;61(3):177-82. doi: 10.1016/s0378-1135(98)00185-0. PubMed PMID: 9631529.
22. Hornberg A, Fernández SR, Vogl C, Vilcek S, Matt M, Fink M, et al. Genetic diversity of pestivirus isolates in cattle from Western Austria. Vet

Microbiol. 2009;135(3-4):205-13. Epub 20080926. doi: 10.1016/j.vetmic.2008.09.068. PubMed PMID: 19019571.

23. Vilcek S, Alenius S, Paton DJ, Mittelholzer C, BelÁK S. Genetic Clustering of Bovine Viral Diarrhoea Viruses in Cattle Farms: Genetic Identification and Analysis of Viruses Directly from Cattle Sera. The Veterinary Journal. 1999;158(1):33-8. doi: https://doi.org/10.1053/tvjl.1999.0363.

24. Choi HM, Moon SY, Yang HI, Kim KS. Understanding viral infection mechanisms and patient symptoms for the development of COVID-19 therapeutics. International Journal of Molecular Sciences. 2021;22(4):1737.

25. Puhach O, Meyer B, Eckerle I. SARS-CoV-2 viral load and shedding kinetics. Nature Reviews Microbiology. 2022. doi: 10.1038/s41579-022-00822-w.

26. Rico-Caballero V, Fernández M, Hurtado JC, Marcos MA, Cardozo C, Albiach L, et al. Impact of SARS-CoV-2 viral load and duration of symptoms before hospital admission on the mortality of hospitalized COVID-19 patients. Infection. 2022;50(5):1321-8. doi: 10.1007/s15010-022-01833-8.
