# Supplementary material for: Contact-number-driven virus evolution: A multi-level modeling framework for the evolution of acute or persistent RNA virus infection
Source: PLoS Comput Biol. 2023 May 30;19(5):e1011173. doi: 10.1371/journal.pcbi.1011173 (PMC10256155; doi:10.1371/journal.pcbi.1011173)
Supplement: Attachment — Submitted filename: PBP_final.pdf [file pcbi.1011173.s003.pdf]

<u>Response to reviewer comments</u>

Junya Sunagawa, Ryo Komorizono, Hyeongki Park, William S. Hart, Robin N. Thompson, Akiko Makino, Keizo Tomonaga, Shingo Iwami and Ryo Yamaguchi

April 17, 2023

We would like to thank the reviewers for their thorough reading of our previously revised manuscript and their constructive comments and suggestions. In this letter, we present the reviewers' comments in black and provide our responses in red. All the changes made in response to the reviewers' comments in PCOMPBIOL-D-22-01431R1 have been incorporated in the revised version of the manuscript. We believe that the new version of our manuscript has been considerably improved by these changes. All coauthors agreed with these changes.

Reviewer #1:

The authors have addressed my main concerns, but there are still some places where the narrative could be better explained and justified:

Reply to Comments:

Thank you for encouraging us through your positive evaluation and for your constructive comments to further improve the quality of our manuscript. In this version, we reply to all the major and minor comments as follows.

【Major comments】

1. Fig. S4 seems to have some other patterns worth mentioning in the main text, including that large variance in daily contacts can select for greater accuracy/lower proliferative capacity (light green dots). The authors might consider moving this figure to the main text, especially since the statement they make in lines 192-195 requires the support of Fig. S4.

Reply to Comments 1 :

Thank you for your suggestion. We have transferred **Fig.S4** to the main text to reflect your comments (see our new **Fig. 4**). As the reviewer suggested, we also added a brief explanation to describe the result highlighting large variance in daily contacts can select for greater accuracy/lower proliferative capacity (Line 190-193).

2. The paragraph beginning on line 210 seems like a strange addition to the results, but would work well in the discussion, perhaps as the first paragraph. In general, the discussion could use a bit more polishing, and the current first paragraph lacks citations and does not add much insight. The paragraphs giving context to previous work are helpful, but it would be even better to end each of those paragraphs by linking the present model results. For example, in the paragraph beginning in line 239, how does examining the mean and variance of contact rates add insight to what has already been learned from comparing large well-mixed populations to small, isolated ones? I'm not sure line 252-253 is true, since lot of models consider seasonal migration patterns of human movement. It would also be more useful to focus on what insight is gained by incorporating stochasticity and extreme rare events, else the paragraph beginning in line 248 reads as unconstructive criticism. One of the strengths of this MS is jointly considering accuracy and replication rates, and that strength is undersold in the two paragraphs referencing past work.

Reply to Comments 2 :

Thank you for your suggestion. As you pointed out, the sentence in the last paragraph of the Result has been moved to the beginning of the Discussion (Line 206-227). The last paragraph of the Results was therefore deleted. In the second and third paragraphs of the Discussion, the advantages of this study based on previous studies and insights into the results were added (Line 236-239 & Line 248-252).

3. In the response to Reviewer 2, the authors mention the key assumption that the duration of the infectious period decreases with cumulative viral load. This assumption is not mentioned until the discussion, and it's necessary to understand the results. The explanation in lines 143-144 does not make this clear, and I suggest adding the text in this response to the first paragraph of the results, because it would greatly clarify this point of confusion:
"…we did not make any assumptions about a trade-off between the accuracy ability of virus () and the proliferative ability of virus (), while it is
assumed that the death rate of virus-producing cells () depends on the proliferative ability of virus () due to viral cytopathogenesis. In contrast, we assumed that the duration of the infectious period decreases with the cumulative viral load, which is a crucial assumption in our study. This is because epidemiological studies have shown that patients with severe disease and a short duration of infection tend to have a high viral load, while those with relatively mild disease tend to have a low viral load [24-26]. Under these biologically reasonable assumptions, the trade-off in the duration of infectious period, as shown in Fig. 1B, arises from our individual-level virus infection model, as described in Eqs.(1- 4)."

Reply to Comments 3 :

Thank you very much for suggesting these changes. We revised our main text to clarify the assumptions that we made for the infectious period (Line 129-134): The durations of the infectious periods $D(V_{\mathrm{total}})$ is assumed to depend on the total viral load. Specifically, the duration of the infectious period decreases with the cumulative viral load. In each parameter combination (i.e., p and ε), $D(V_{\mathrm{total}})$ is calculated by Eqs.(5-6) based on the virus infection model [Eqs.(1-4)] with other fixed parameters (see **Table S1**). The calculated infectious period for each parameter pair is described in **Fig.1B**.

【Minor comments】

4. Line 128-130: Would be helpful to break this sentence down and go through the logic more gently.

Reply to Comments 4 :

Thank you for your helpful comments. In the revised version, we've briefly explained what we assumed in our model (Line 127-131).

5. Line 140: Suggest omitting "mainly reflecting into" for clarity

Reply to Comments 5 :

We have deleted the words "mainly reflecting into" at Line 125 in accordance with the indications given in this comment.

6. Line 135: Would be good to start with something like what is currently in the methods (lines 378-389), so that readers have some idea where the results are coming from.

Reply to Comments 6 :

We totally agreed with this comment and added sentence as follows (Line 135-137): To evaluate how the proliferative ability and accuracy of replication of viruses are optimized to increase the transmission potential, we assumed that environments are characterized by the number of contacts per day.

7. Figure 1 caption: The new figure is very helpful but it would be even clearer if the caption referenced mathematical terms, for example making line 747-8 read "The probability of infection (rho) of susceptible individuals from an infected hosts depends on their viral load (Vi(t))." Likewise, line 750 could read "…the contact numbers (c(t)) are assumed to follow…"

Reply to Comments 7 :

Thank you for your suggestions. We revised the caption accordingly (Line 740-743).

8. The explanation from lines 192-208 was a little hard to follow because the text refers to unlogged contact rates and the figures show logged contact rates. Maybe keep the log scale in Fig. 3 but replace labels with unlogged values?

Reply to Comments 8 :

Thank you for pointing this out. We changed the labels in **Fig.3**.

9. Lines 345-347: suggest omitting these sentences—no model (or experiment) will ever fully reflect the detailed physiologic processes occurring within the host in a natural setting.

Reply to Comments 9 :

As this comment suggests, we've deleted the sentence (Line 343-345).

10. Line 356—Start a new paragraph with "Our mathematical model for population transmission…" [Omit the "Another modeling limitation"—no need to apologize for not considering everything in a single study.]

Reply to Comments 10 :

We have dropped the expression and started a new paragraph as suggested (Line 351).

11. Lines 371-375—Split up this sentence for clarity.

Reply to Comments 11 :

Thank you for your helpful comments for better understanding. With regard to Line 353-356, the relevant text has been modified as follows; If entire chains of transmission are considered, it may be necessary to consider stochastic effects that may lead to particular viral phenotypes failing to become established in host populations. This would happen particularly in the region of a low-density contact history (the mean contact number from 0.01 to 1) for which $R_{TP} \approx 1$ [59].
